# Gendered Attitudes Toward Corporal Punishment: Implications for Prevention of Mental Health Problems in Youth

**DOI:** 10.3390/healthcare13233053

**Published:** 2025-11-25

**Authors:** Miroslav Rajter, Milani Medvidović

**Affiliations:** 1 Faculty of Law, University of Zagreb, 10000 Zagreb, Croatia; 2 Elektrotehnička Škola—Split, 21000 Split, Croatia

**Keywords:** corporal punishment, gender differences, attitudes, mental health, youth wellbeing, violence prevention, vignette-based scale

## Abstract

**Background**: Corporal punishment is a form of violence that poses long-term risks to children’s mental health and wellbeing. Understanding the attitudes that justify such practices is essential for designing preventive and health promotion interventions. Previous research suggests gender differences in these attitudes, yet the extent and nature of these differences remain unclear. Objective: This study examined gender-related differences in attitudes toward corporal punishment and their implications for youth mental health promotion. Participants and Setting: The study involved 582 university students aged 18 to 40, with a mean age of 22 years. Participants were from various fields of study and were surveyed online. **Methods**: The Short Situational Scale of Attitudes towards Corporal Punishment (SSS-CP) was developed for this study, depicting hypothetical conflicts between parents and children, culminating in corporal punishment. A quasi-experimental design was used, varying the gender of the participant, parent, and child. Data was analyzed using ANCOVA, controlling for previous experience of corporal punishment. **Results**: Physical punishment was more justified when the participant was male (6% of criterion variance), when the perpetrator was a female parent (1.3%), and when the child was male (1.8%); however, no significant interaction effects were found. Previous experience with corporal punishment also predicted more approving attitudes toward its use (1.7% of criterion variance). **Conclusions**: Gender differences in the justification of corporal punishment highlight how social norms shape the acceptance of violence and, consequently, the normalization of behaviors linked to poorer mental health outcomes in youth. Prevention and health promotion programs should integrate gender-sensitive components that address beliefs about violence, foster emotion regulation, and reduce the intergenerational transmission of harmful disciplinary practices.

## 1. Introduction

Child abuse is one of the key determinants of mental health and emotional wellbeing during childhood and later in adulthood [1]. In this manuscript, the terms *corporal punishment* and *physical punishment* are used interchangeably to denote disciplinary practices involving physical force with the intention of causing pain or discomfort. Among the forms of violence against children, corporal punishment has been consistently linked to higher rates of anxiety, depression, aggression, and impaired emotional regulation in youth, underscoring its relevance to mental health prevention frameworks. International public health frameworks increasingly emphasize the elimination of corporal punishment as part of child rights fulfillment and mental health promotion. Initiatives such as the WHO’s *End Corporal Punishment Initiative* [2] and UNICEF’s global parenting and violence-prevention programs underscore that physical punishment constitutes a modifiable risk factor for poor psychological outcomes. Situating attitudes toward corporal punishment within these global agendas highlights the need for evidence that informs prevention and supports positive parenting practices. Research on attitudes toward corporal punishment is therefore crucial, as parents’ use of physical punishment is strongly predicted by their attitudes. According to Gershoff [3], there is a larger possibility of this type of violence being used when one believes that positive effects will outweigh negative ones. Holden and colleagues [4] emphasize that justifying physical punishment predicts its frequency and intensity. According to Rajter et al. [5], sentiments toward physical punishment of children are not different among mothers and fathers. This paper also highlights findings from a Croatian survey of parents, in which approximately one-third expressed support for corporal punishment as a disciplinary method. Pećnik and Brunnberg [6] analyzed justifying violence regarding the gender of the parent and the child and found that physical punishment is more strongly denounced when the perpetrator is the father, and the victim is a girl. As the authors explain, punishing boys is considered more socially acceptable than punishing girls. Additionally, professionals often express concern that physical punishment of girls may overlap with or mask sexually intrusive behaviors, reflecting heightened sensitivity to the gendered risks girls face in broader patterns of maltreatment [6]. These norms also intersect with mental health, as tolerance for violence may reduce empathy and increase emotional disengagement, both of which are associated with lower wellbeing.

Rajter [7] notes that monitoring of violence in families is not systematic. Small and non-representative samples, as well as varying methodological approaches, hinder the ability to track changes in the prevalence of violence against children in families. The National Research Council [8] highlights the problem of unclear definitions of unacceptable and harmful parental behavior, differences in defining physical punishment and abuse for scientific, legal, and clinical purposes, as well as uncertainties in the interpretation of procedures regarding age, gender, nationality, relationship with the perpetrator, and context. Despite these definitional challenges, the global public health community, including WHO and UNICEF, increasingly recognizes corporal punishment as a modifiable risk factor for mental health problems in childhood and adolescence.

Previous studies on attitudes towards physical punishment of children have mainly used questionnaires to measure attitudes towards physical punishment at a general, conceptual level. One drawback of such scales is social desirability bias of the respondents, which produces responses that do not align with epidemiological evidence showing high prevalence rates of physical punishment [7]. To improve the ecological validity of such assessments and better inform mental health prevention programs, it is important to capture how individuals justify violence in realistic interpersonal contexts. To address this gap, this paper will examine attitudes towards physical punishment of children in a specific event (a vignette) in which a hypothetical situation of conflict between parents and a child ending with physical punishment is presented. The aim of the vignette is to contextualize the situation so that respondents with more positive attitudes towards physical punishment can justify the parent’s behavior, thereby reducing social desirability bias. Additionally, previous research has rarely examined gender differences simultaneously across the participant, parent, and child, leaving a gap in understanding how gendered social norms shape the justification of violence. By adapting a vignette-based, context-specific measure, this study addresses both the methodological limitations of abstract attitudinal scales and the conceptual gap in cross-gender comparisons.

This study was conducted in the Croatian context. While certain national characteristics, such as legal regulation, culture, and behavioral norms, are specific to Croatia, the phenomenon-related characteristics are applicable to the international context, both in terms of content and methodology. Its conceptual and methodological implications align closely with international violence-prevention and mental-health promotion frameworks, contributing to global evidence despite regional specificities.

In line with contemporary public health approaches, this study situates attitudes toward corporal punishment within the broader framework of violence prevention and youth mental health promotion. By identifying gender-related patterns in the justification of violence, it aims to inform targeted psychoeducational and policy interventions that foster safe, supportive, and mentally healthy environments for children and adolescents.

### 1.1. Physical Punishment and Physical Abuse

Any form of discipline that uses physical force with the goal of inflicting pain or discomfort is referred to as corporal punishment. The term “corporal punishment” most often refers to striking children with a hand or other item, but it can also apply to other methods such as throwing or shaking children, pinching, scratching, pulling their hair, kicking, burning, putting them into awkward positions, or forcing them to swallow substances [9]. From a health perspective, these practices are associated not only with physical injury but also with psychological consequences such as increased emotional distress, heightened physiological stress responses, and long-term risk for anxiety, depression, and antisocial behavior.

It is challenging to come up with a clear-cut definition for this type of domestic violence that is commonly acknowledged [10]. This form of domestic violence comprises a variety of actions that differ in severity, frequency, and method. Physical punishment can also be defined as behavior undertaken to punish a child that, if directed at an adult, would be considered an illegal assault [11], but also as the use of force with the intention of causing pain, not injury, with the aim of controlling or correcting a child’s behavior [12].

Physical abuse and physical punishment are often compared. According to Velki and Bošnjak [13], physical abuse of children is a type of domestic violence that is simply and completely defined as injuring a child physically. But much like with physical punishment, there are many different and nuanced definitions of physical abuse. The term “physical abuse” is defined differently depending on the cultural, social, legal, and medical factors that are particular to that country [14]. Despite numerous attempts to establish a widely accepted definitions of physical punishment and physical abuse, the problem of contextualization and operationalization persists. Some researchers conceptualize physical abuse and physical punishment as two points on a continuum. The main difference they cite is intensity, which refers to the severity of violent behavior, with the presence of physical injury being a common indicator. Behavior that is assessed as low risk (not causing or resulting in minor physical injury) is considered punishment. Conversely, behavior assessed as high-risk (resulting in serious physical injury) is characterized as abuse [10]. In addition to intensity, frequent physical punishment may also constitute abuse, distinguishing chronic patterns from isolated incidents [8]. These criteria do not necessarily indicate the seriousness of the violence and overlook the psychological consequences for the victim [10]. Slapping, hitting with an object, or pushing are just a few examples that may not necessarily have visible consequences, but still pose a risk to a child’s development. In addition to intensity and frequency, the definitions of physical punishment and abuse often includes the perpetrator’s intent. According to Straus and Donnelly [12], physical punishment is applied with the intention of causing pain, not injury, to control or correct a child’s behavior. However, this criterion is also debated, as Pećnik [10] notes that the use of violence is sometimes more related to the parent’s emotional state than to control the child’s behavior. Azar [15] offers another explanation, stating that physical punishment and physical abuse are different types of parental behavior, and physical punishment is interpreted as the ultimate point on the continuum of “normal” parental behavior, unlike abuse.

In any case, any continuum separating “discipline” from “abuse” is clinically and ethically problematic, as both can contribute to toxic stress and maladaptive coping. The current body of literature suggests that physical punishment is associated with harm to physical and mental health, impaired cognitive and socio-emotional development, atypical brain development, as well as behavioral problems, poor moral internalization, increased aggression and greater acceptance and use of violence in society [1]. Prospective and cross-sectional studies consistently demonstrate that corporal punishment predicts elevated risks for anxiety, depressive symptoms, externalizing behavior, substance misuse, suicidal ideation, and impaired emotion regulation across childhood and adolescence. Neurobiological research also suggests associations with altered stress-response functioning, heightened reactivity to threat, and atypical development of brain regions involved in emotional processing. Viewing corporal punishment through a public health lens enables the integration of prevention, early intervention, and education into mental health promotion systems.

### 1.2. Attitude Towards Corporal Punishment

Although corporal punishment has been prohibited in the Republic of Croatia since 1999 by the Family Law, the Law on Protection from Domestic Violence, and the Criminal Code, a certain number of parents still physically punish their children [7]. In a 2006 study, Pećnik found that 93.4% of students had experienced some form of physical punishment during childhood. In her book “Intergenerational Transmission of Child Abuse” [10], Pećnik presents a study conducted on primary and secondary school children. Between 71% and 86% of children had experienced some form of physical violence from their parents at least once in their lifetime. Valuable yet concerning data were obtained from the research by Ajduković and colleagues [16], collected as part of the international BECAN study (Balkan Epidemiological Study on Child Abuse and Neglect), according to which 56.1% of fifth graders (11 y/o), 68.4% of seventh graders (13 y/o), and 72.3% of second-year high school students (16 y/o) had been physically punished during their lifetime.

One potential explanation for the results obtained relates to attitudes. Although the definition of attitudes is discussed, for the purpose of this paper we can use the definition proposed by Zanna & Rempel [17] where attitudes are seen as the categorization of a stimulus object along an evaluative dimension based upon, or generated from, three general classes of information: (1) cognitive information, (2) affective/emotional information, and/or (3) information concerning past behaviors or behavioral intentions. Attitudes toward corporal punishment are among the most significant predictors of parents’ use of physical punishment. Individuals who apply physical punishment most often have positive attitudes towards it [18]. Gershoff [3] and Gagné et al. [19] are among the numerous researchers who note that individuals with a positive attitude believe that the use of corporal punishment is not associated with harmful consequences for the child’s development. Gagné et al. [19] further explain that individuals who believe that punishment does not cause or rarely causes physical injuries are those who most strongly support violence. Attitudinal tolerance toward physical punishment can thus normalize coercive family dynamics, reducing emotional safety and undermining children’s mental wellbeing. For public health professionals, these beliefs represent modifiable targets for preventive interventions.

In the study by Rajter et al. [5], a third of parents reported positive attitudes toward corporal punishment, and the majority believed that corporal punishment is a widespread practice. Only 17.7% of participants expressed a distinctly negative attitude and disapproval. Trbus et al. [20] conducted a study with a sample of 5076 participants. According to the results, 41.8% of young people, 31.9% of parents, 32.3% of school staff, and 6.7% of kindergarten staff believed that “it is justified to hit a small child on the buttocks to teach them what they should not do.” Additionally, 42.5% of young people, 22.8% of parents, 20.6% of school employees, and 3.7% of kindergarten staff agreed with the statement that “an occasional slap will not harm a child’s development.” It is also important to note that 16.6% of young people state that they will use physical punishment as parents when the child deserves it, while 20% are unsure. These findings indicate a lack of awareness among young people, parents, and professionals about the potentially harmful consequences of physical punishment, as well as the insufficiently developed attitudes of young people toward the physical punishment of children. This developmental period provides an opportunity to expose young people to preventive and educational programs to help them critically approach traditional patterns advocated and modelled by the environment. As part of the project “From Policy to Reality—Changing Attitudes and Practices from Corporal Punishment to Child Protection Measures,” Brave Phone [21] conducted a study aimed at examining attitudes towards physical punishment of children. 52% of participants believe that physical punishment generally should not be used, but they justify it in certain situations. 7% believe it can be used in situations where it might be effective, 2% are unable to assess, and only 39% recognize the harmful consequences and consider physical punishment unjustified. Thus, attitudes towards corporal punishment are considered key antecedents of child maltreatment and a factor that should be a part of all prevention programs, e.g., [22], making them an important target for promoting children’s mental health and emotional wellbeing. Given that young adults may become future parents, understanding their beliefs provides a window for early prevention. Challenging these justifications may strengthen empathy, emotional regulation, and non-violent conflict resolution, all protective factors for mental health.

### 1.3. Gender Differences in the Attitude Towards Corporal Punishment of Children

In researching attitudes toward corporal punishment, certain researchers have focused on gender differences among perpetrators, victims, and observers. In the study by Pećnik et al. [23], which examined public beliefs about correct parental behaviors, no significant gender differences were found. Men and women justified physical punishment of children to a similar extent. In the study by Rajter et al. [5], a statistically significant difference in attitude towards physical punishment of children was observed between men and women (men show a more positive attitude towards physical punishment than women); however, considering the effect size, the difference is small. Fathers and mothers in the mentioned study have equally expressed attitudes towards physical punishment of children. Despite the consistent results of Croatian studies, some foreign research indicates that men are more likely to justify physical violence. Chiocca [24] in her work cites a study by Child Trends in the USA, which found that 76% of men and 65% of women aged 18 to 65 believed that a child needs to be “well hit” occasionally. Flynn [25] conducted a study on students and reports that men have more positive attitudes towards physical punishment than women, while Deater-Deckard et al. [26] conducted a longitudinal study which found that male adolescents justify violence more than female adolescents.

Across studies examining gender differences in children’s exposure to physical punishment, results predominantly indicate higher exposure among boys. This finding is confirmed by Lansford et al. [27]. In a sample of 1417 children, they found that 54% of girls and 58% of boys had experienced physical punishment at home in the last month. Consistent with these findings, Akmatov [28] cites a UNICEF study in 29 countries which found that boys are at greater risk of physical abuse. Gershoff [3], in a meta-analysis, cites a series of studies consistent with these findings. Some researchers, such as Meeks Gardner et al. [29], have linked this difference to boys’ greater aggressiveness and their engagement in behaviors perceived as “provocative”. However, Gershoff [3] questions the assumption that parents use corporal punishment simply because boys behave more aggressively. Longitudinal studies show that corporal punishment itself contributes to later aggression and emotional dysregulation, indicating that violence by parents is not only a reaction to the child’s behavior but also a factor that increases problematic behavior over time. That boys are more often victims of violence is also confirmed by the attitudes of young people. In the study by Trbus et al. [20], it is evident that young people believe that young men are more often victims of violence than young women. 34% estimate that almost all girls have experienced being hit by someone in the family, while 42.1% estimate that almost all young men have experienced being hit by someone in the family. Despite the dominance of research highlighting that boys are more often victims of physical punishment, Ajduković et al. [16] obtained results that deviate somewhat from these findings. The study involved fifth and seventh-grade primary school students and second-grade high school students. Among fifth-grade students (approximately 11 years old), it was shown that boys were more exposed to physical punishment, in the seventh grade (approximately 13 years old) there were no significant gender differences, while in the second grade of high school (approximately 16 years old), girls were more often exposed to physical punishment. As a possible explanation, Caffaro [30] explains that as children grow up, power relations are defined, and if it involves brothers and sisters, violence against boys decreases. Such findings highlight gender socialization as a mechanism influencing emotional expression, aggression, and perceived legitimacy of violence as factors closely related to mental health outcomes.

Men’s higher endorsement of corporal punishment may reflect traditional masculinity norms emphasizing control and toughness, while women’s lower acceptance might relate to nurturing roles and empathy expectations. These gendered schemas also shape the emotional consequences of both perpetration and victimization, potentially influencing long-term wellbeing.

### 1.4. Experience of Violence in Childhood

The relationship between experiencing violence in childhood and attitudes towards physical punishment is complex and multifaceted. The intergenerational transmission of violence is a widely recognized framework in explaining child abuse [31]. Numerous studies have found that a significant number of parents who were physically punished in childhood resort to similar behaviors in parenting and tend to perceive such behaviors as less harmful and serious [32]. This correlation between the experience of physical punishment and lower self-esteem in children, as well as weaker problem-solving skills, is notable. Low self-esteem and low self-efficacy are linked to the later use of violent methods towards one’s own children [33].

Most research in this area confirms the significance of childhood violence experiences as predictors of approving attitudes towards physical punishment. Adolescents who were physically punished during childhood are more likely to approve of this type of such practices than those who were not [26]. Buntain-Ricklefs et al. [34] report that parents who experienced physical punishment themselves view such methods as more justified and appropriate compared to parents who did not experience such punishment in childhood. Among parents who experienced slapping or spanking in childhood, a significant percentage continue to approve of it in adulthood. Growing up in such environments, violence can become normalized as a way of solving problems and later as an appropriate educational method [35]. Some parents idealize their own parents and childhood experiences. Main and Goldwyn [36] describe idealization as a defense mechanism developed to cope with the trauma of punishment and abuse. Buljan Flander [37] explains how a child develops an internal working model according to which adult relationships are formed consistent with those in childhood. The attachment pattern acquired in childhood later develops with one’s own child. Other explanations include the Social Learning Theory, which suggests that aggressive actions are learned through reinforcement and punishment. Genetic factors and the Multifactorial Theory, which integrates various approaches (socioeconomic status, characteristics of parents and children, cultural values, stress), are also potential explanations [38]. This cycle maintains risk for mental health problems across generations, including trauma symptoms and maladaptive emotion regulation.

However, not all research supports this correlation. In the study by Bower and Knutson [39], a group of young people who had experienced physical punishment in childhood condemned this parenting method, showing increased awareness of potential negative consequences. As noted by Pećnik [10] and Rajter [14], many parents with a history of childhood violence do not perpetrate violence against their children. To clarify these mixed findings, recent research investigates factors that differentiate parents who perpetuate the intergenerational transmission of violence from those who do not continue to use violent methods.

### 1.5. Present Study

In the context of violence prevention and youth mental health promotion, understanding the cognitive and gender-related foundations of attitudes that normalize corporal punishment is essential. This study introduces the Short Situational Scale of Attitudes toward Corporal Punishment (SSS-CP), a vignette-based instrument designed to measure context-specific justifications of corporal punishment. We examine the effects of participant, parent, and child gender on the justification of corporal punishment while controlling for participants’ own experiences of being physically punished. By identifying gendered patterns of moral reasoning about violence, this study contributes to evidence-based strategies for mental health promotion and prevention of family violence.

The study’s focus on ‘moral reasoning’ reflects the idea that attitudes toward corporal punishment involve evaluative judgments about right and wrong within interpersonal conflict. Vignette-based methods are well suited for eliciting such judgments because they place respondents in morally charged situations that require evaluation of intentions, consequences, and relational expectations.

Based on previous research, we hypothesized that (1) men would show more approving attitudes toward corporal punishment than women; (2) corporal punishment would be more justified when the child in the vignette is male; and (3) individuals with personal histories of corporal punishment would endorse more approving attitudes. We also examined whether the gender of the parent in the vignette was related to attitudes, but given the mixed findings in the literature, we treated this as an exploratory, non-directional expectation. Since the literature on the interaction effects is scarce, we proposed null hypotheses.

## 2. Method

### 2.1. Participants

The study was conducted with university students in Croatia. Participants were recruited through university mailing lists and established student groups across several faculties in Croatia. This non-probabilistic recruitment approach was selected to ensure broad disciplinary coverage and to obtain a sufficiently large and diverse student sample suitable for the aims of the study. To ensure internal validity, participants were randomly assigned to one of four vignette conditions, resulting in an unbiased distribution of individual characteristics across experimental groups. Such random allocation is a key requirement for valid estimation of the effects of manipulated factors, thereby reducing potential concerns related to the use of convenience sampling. This approach is consistent with standard methodological practice in experimental vignette studies and attitudinal research. A total of 863 participants initially took part in the study. However, after the exclusion of inadequate responses and to ensure an equal number of male and female participants using the random sampling technique, the final sample consisted of 582 individuals. Gender balancing was conducted only at the level of final sample selection and did not affect the prior random assignment to vignette conditions. The final sample included 291 male participants (50%) and 291 female participants (50%). The age range of the participants was from 18 to 40 years, with a mean age of 22 years (SD = 2.31). Although some participants, based on age, were non-traditional students, we retained these responses because such students increasingly represent a meaningful proportion of university populations, and because random assignment to vignette conditions ensured that age outliers did not systematically bias group comparisons. The fields of study of the participants were diverse, including social sciences (27.5%), technical sciences (37.3%), humanities (11.2%), biomedical sciences (10.1%), natural sciences (6.5%), interdisciplinary studies (3.1%), biotechnical sciences (2.9%), and arts (1.4%). A significant proportion of participants, 387 individuals (66.5%), reported having experienced corporal punishment during childhood, with the age at last experience ranging from 2 to 24 years.

### 2.2. Measures

Before completing the questionnaire, participants were asked to respond to sociodemographic questions such as gender, age, and field of study. Additionally, participants were requested to answer whether they had experienced corporal punishment during their childhood and, if so, their age at the time of the last occurrence.

#### Short Situational Scale of Attitudes Towards Corporal Punishment

For the purposes of this research, the Short Situational Scale of Attitudes toward Corporal Punishment (SSS-CP) was developed by the authors of this study. The SSS-CP includes a description of a specific event (vignette) depicting a hypothetical conflict situation between a parent and a child, culminating in corporal punishment. The vignette was developed through an iterative process involving expert consultation and pilot testing with a small group of graduate students (n ≈ 20). The scenario was selected because it reflects a realistic parent–adolescent conflict involving transgression, verbal escalation, and physical punishment. Gender was manipulated solely through parent labels (mother/father) and child names commonly recognized as male or female. The questionnaire contains four vignette variations in which the gender of the child and the parent varies, allowing examination of differences of gender-based differences in the justification of corporal punishment. Parent gender was indicated by the terms “mother” or “father”, and the gender of a child is indicated by the unambiguous name typical for males or females. The scale consists of nine statements reflecting the three fundamental components of attitude (cognitive, emotional, and behavioral) towards corporal punishment of children. Agreement with each statement is expressed on a Likert scale ranging from one to five, where one signifies “strongly disagree” and five indicates “strongly agree”. The scale score is computed as the average response to all items. The reliability coefficient, Cronbach’s alpha, was high, amounting to α = 0.95 (α for each condition of participant, parent and child gender was 0.95, except for female participants where it was 0.94). As the instrument was newly developed, a principal component analysis conducted on the full sample (N = 582) after data collection confirmed a unifactorial structure. The scale is included in the Appendix A of this paper.

Because the scale includes a single vignette, its contextual specificity may limit generalizability to other types of disciplinary incidents. Future adaptations should incorporate multiple scenarios varying in child age, behavior severity, relational dynamics, and contextual triggers to strengthen content validity.

### 2.3. Procedure

The questionnaire was administered as an online form. Following approval from the Ethics Committee of the Department of Psychology at the University of Zagreb Faculty of Croatian Studies, the link to the questionnaire was shared in student groups on Facebook and distributed to university mailing lists. Upon accessing the link, participants were presented with instructions that included a description of the research purpose and a note that participation was anonymous and voluntary. It was also stated that the collected data would be analyzed and presented at the group level, used solely for scientific research purposes, and that participants could withdraw from the study at any time. By clicking the “Next” button, participants were considered to have consented to participate in the study. In case of uncertainties or additional questions, participants could contact a student researcher via the email address provided. After reading the instructions, each participant was randomly presented with one of four vignette variants (SSS-CP) along with sociodemographic questions and questions about experiences of corporal punishment in childhood. Completing the questionnaire took approximately 5 min, and data were collected from 1 June to 21 June 2021.

## 3. Results

All analyses were performed using R 4.1.2 [40] with *tidyverse* 2.0 package [41]. The full R quarto code for the analysis, including the link to the github repository with the data, can be found in the Supplementary Materials.

### 3.1. The Structure and Invariance of SSS-CP Scale

Confirmatory factor analyses were performed using *lavaan* 0.6-13 package [42]. The presumed structure for the Attitudes towards Corporal Punishment—Short Situational Scale was a single-factor solution. The model was estimated using the diagonally weighted least squares estimator (DWLS), which is a robust method appropriate for ordinal variables [43]. The model showed an excellent theoretical fit (χ^2^ = 27.99; df = 27; *p* = 0.411, CFI = 1.000, TLI = 1.000; RMSEA = 0.008 [0.000–0.034]; SRMR = 0.032), indicating that the data fit the proposed single-factor solution.

Measurement invariance was assessed for participant gender, parent gender and child gender (Table 1). The primary method of the invariance testing was to create models for each group (*group* parameter in *cfa()* function) and then to use constraints for loadings and intercepts. The invariance was assessed by the difference in χ^2^ provided by *anova()* function and changes in CFI, RMSEA and SRMR.

The results show that all models demonstrated good fit. Regarding the invariance for the participant’s gender, the difference in the models is statistically significant based on the $\chi^{2}$ tests, the difference in CFI parameter is less than 0.002, which is the most strict cut-off value as recommended by Kline [44]. Regarding the parent’s and child’s gender the models show that there is no statistically significant difference between each model. We conclude that the SSS-CP demonstrates functional invariance across participant, parent, and child gender.

The scale score were calculated as the mean of all items. This scoring procedure included reverse-coding items 5, 6, 7 and 9. The Cronbach α reliability of the scale was 0.949.

### 3.2. Item and Scale Descriptives

The descriptive statistics for the items and the total scale score are shown in the Table 2. The descriptive statistics were calculated using the *reflexR* 0.9 package [45].

The results show that the answers of participants are on average close to the theoretical midpoint of the scale, indicating good sensitivity of the instrument. All analyzed variables deviated significantly from the normal distribution. Although the absolute values of skewness were relatively small, indicating good distribution of responses across the scale, values of kurtosis reflected a somewhat “flatter” distribution, which is to be expected when the attitudes toward a controversial topic are measured.

### 3.3. Gender Differences in Attitude Towards CP

The final analysis focused on testing the differences related to participant, parent and child gender. Three-way ANCOVA was performed with main effects of participant, parent and child gender entered as main effects. All interaction effects were analyzed and the previous experience with corporal punishment was included as a covariate. The analysis was used Type III sum of squares method and was conducted with the *jmv* 2.4.9 package [46]. Table 3 and Table 4 show the model summary and the descriptive statistics for significant effects.

The results show that all three main effects are statistically significant and none of the interaction effects are statistically significant. Thus, the attitudes towards corporal punishment are more approving when the participant is male, when the child is male and when the perpetrator is female. In terms of effect size, the most important effect is participant gender that explains 6.8% ($\eta^{2}$) of the attitudes towards corporal punishment with males having more approving attitudes than females. The other two main effects explain less than 2% of the dependent variable and show that the participants have more approving attitudes towards corporal punishment when the perpetrator was female, and the child was male. The interaction effects were insignificant and in terms of effect sizes explained only trivial proportions of variance.

## 4. Discussion

Corporal punishment remains a controversial topic, although its negative effects on the mental health and emotional wellbeing of children and adults are well established [1]. Research consistently associates exposure to physical punishment with higher rates of depression, anxiety, aggression, substance misuse, and poor emotional regulation in adolescence and adulthood [1,3,47,48]. By framing the results within a mental health promotion context, our study highlights that attitudes justifying corporal punishment sustain the normalization of psychologically harmful behaviors. These findings align with international meta-analyses showing consistent links between corporal punishment and adverse mental-health outcomes across cultures, including increased internalizing symptoms, aggression, and dysregulation [3,47,48]. Evidence from large prospective studies further confirms that corporal punishment is a predictor of later anxiety, depression, and behavioral difficulties across developmental contexts [47,48]. Changing attitudes represents a preventive intervention point that may reduce both acceptance of violence and the risk of future perpetration, as well as psychological distress associated with growing up in violent households.

While the number of countries who have introduced a ban on corporal punishment of children is growing, the prevalence rates remain relatively high. Traditional measurement of attitudes towards corporal punishment of children is based on phenomenon-related content with items such as “It is justified to spank your child if it does something wrong”. This kind of measurement, while common in the field of measurement of attitudes, has the drawback that the items’ context is abstract, and it is more susceptible to social desirability.

In this study, we constructed a new vignette-based scale. The primary motivation for developing this scale was to produce an instrument that would have such calibration that the sample mean would be close to the theoretical mean and to allow enough dispersion to improve the statistical detection of effects. While the scale metrics provide enough data to infer the validity of the scale, this approach introduces challenges. The text of the vignette is strongly culturally dependent on several aspects. Not only was the construction of the text based on the Croatian context, but the crucial part of the text is also the provision of the “justification” of the parent’s violent reaction. For example, during the construction, the curse words that the child says to the parent were selected to be as applicable as possible regardless of the child’s gender, but also applicable to the parent, regardless of the gender and with the same intensity. While the semantic analysis of the used text provided enough justification for the selection of these curse words, there is a lack of data on the gender-based appropriateness and intensity. In the context of our results, this provides a future opportunity for the improvement of the scale; however, the issues posed here are also time-dependent because, in the context of our scale, both descriptions of the “transgression of the child” and the curse words intended to provide the justification for the violent reaction change their meaning over time. This is not specific to this scale because the content validity is time-variant due to the changing nature of the words and their meanings.

The main goal of this study was to examine gender-related attitudes towards corporal punishment. Previous studies have indicated a more approving attitudes in men compared to women or no statistical difference. This study confirmed the difference with a 7.3% of the variance in attitudes being attributed to participant’s gender. This pattern is consistent with research showing that masculinity norms emphasizing control, authority, and instrumental responses to conflict correlate with higher acceptance of corporal punishment. Cross-cultural evidence suggests that male-identifying individuals are more likely to interpret violence as a functional disciplinary tool, whereas female-identifying individuals tend to emphasize empathy and relational processes, which reduces endorsement of physical punishment. The explanations for this finding are complex, and this phenomenon can be approached from a multitude of perspectives. The results on the prevalence of corporal punishment show that boys are more exposed than girls at an earlier age, e.g., [49], which can lead to the normalization of violence as a response mechanism. Another perspective concerns socialization and gender norms where men are more likely to assume the traditional roles emphasizing dominance, control and physical strength. For example, Valls et al. [50] found that teenagers associate hegemonic model of masculinity with attractiveness. The third perspective is a psychological perspective where men show higher importance of promotion values that include power, prestige, and success [51] or, for example, higher Machiavellianism, psychopathy and narcissism [52] or Machiavellianism and psychopathy in the Croatian sample [53]. Acceptance of violence as a corrective measure may thus reflect internalized norms that weaken mental health through suppression of empathy and avoidance of emotional vulnerability.

The second finding is that the violence is more justified when the perpetrator is a woman, and this effect was small with 1.5% of the variance in attitude being attributed to the parent’s gender. One possible explanation is that maternal aggression is often interpreted within a caregiving frame, where mothers’ actions are viewed as emotionally driven or corrective rather than punitive. Research on gendered socialization suggests that the same behavior is judged differently depending on the gender of the perpetrator, with violence by fathers perceived as more threatening and violence by mothers more easily normalized. Such interpretive biases may obscure the emotional and mental-health impacts of maternal aggression on children. It can thus be that the violence committed by mothers is more often perceived as a function oriented towards upbringing or self-defense compared to the violence committed by men. However, as already described regarding the participant gender, this issue is complex, and no single explanation can encompass the phenomenon. This bias may obscure the emotional impact of maternal aggression, particularly for male children, who are socialized to minimize emotional pain.

The third finding was that the violence was perceived to be more justified when directed toward male than female children, with 2% of variance in attitudes attributed to child’s gender. While previous gender-based explanation can be applied here, two other aspects might be included. The first aspect is that a cultural norm expects the parents to raise their children in their gender roles, which assumes the explanation of a harsher discipline for boys. The other aspect is related to the age of the child. In our scenario the child is aged 16 and thus the power dynamics is not the same if the child was of a younger age. It is possible that the power balance between a parent and a 16-year-old boy is perceived to be more equal than with a 16-year-old girl.

The tests for interaction effects showed no significant results. Gershoff [3] indicated inconsistent findings regarding the application of corporal punishment and the interaction of the parent’s and child’s gender. These findings suggest that attitudes towards corporal punishment operate independently of the combinations of participant, parent, and child gender.

Our results also indicate the main effect of previous experience with corporal punishment. This effect is supported by the previous literature where corporal punishment is seen as more justified among people who were physically punished. This also identifies an opportunity for preventive intervention: university-age populations, many of whom are future parents, are an ideal target group for psychoeducational programs promoting non-violent discipline and emotional competence.

As an unexpected finding, our study showed that the oldest age our participants report that they experienced corporal punishment is 24. While this was not the primary goal of our study, further analysis shows that 4.5% of participants experienced corporal punishment at age 18 or older. From the phenomenon perspective, this introduces a specific population where legal frameworks do not recognize them as children and support services for child protection do not recognize them as potential clients. However, some university students continue to live within their primary families and are financially dependent on parents, family dynamic may remain unchanged into young adulthood. As one of the implications of this study, we can accentuate the need for studies within this population who are in specifically vulnerable position and in a high risk for their mental health and safety within their primary family.

Although vignette-based methods reduce some forms of social desirability bias, they cannot eliminate it entirely. Participants may still provide judgments that align with perceived social norms rather than personal beliefs, and the written nature of the vignette limits emotional immersion. Future research could integrate qualitative follow-ups, mixed-method designs, or implicit measures to further validate attitudinal responses.

From a prevention perspective, interventions addressing beliefs about corporal punishment can function as early mental-health promotion tools. Programs for university students should include structured modules on non-violent discipline strategies, emotional-regulation skills, and the psychological consequences of violence. Public health campaigns can integrate gender-sensitive components that challenge norms equating masculinity with toughness or control and promote caregiving as compatible with emotional competence. In educational settings, evidence-based parenting programs such as positive discipline programs [54], Triple P [55], or WHO’s Parenting for Lifelong Health [56] can be adapted to directly confront justificatory beliefs and strengthen empathy, conflict-resolution skills, and mental-health awareness. Embedding these approaches across community, healthcare, and school systems can reduce the normalization of violence and support healthier developmental environments for youth.

Overall, the study contributes to international efforts aimed at reducing corporal punishment by demonstrating how gendered norms and personal histories shape moral reasoning about violence. Integrating these insights into public-health, educational, and policy contexts can support the creation of safer interpersonal environments and promote psychological wellbeing across generations.

## 5. Conclusions

This study examined gender-related differences in attitudes toward corporal punishment using a vignette-based instrument designed to minimize social desirability bias. The results showed that men, participants exposed to corporal punishment in childhood, and those evaluating scenarios involving male children or female perpetrators were more likely to justify its use. These findings indicate that gender norms and personal experience shape the moral reasoning that sustains tolerance for corporal punishment.

From a health perspective, these attitudes represent a psychosocial risk factor. Acceptance of violence in parent–child relationships can normalize aggression, weaken empathy, and contribute to emotional dysregulation, processes associated with poorer mental health outcomes across the lifespan. Addressing these beliefs is therefore not only a matter of social policy but also an essential component of youth mental health promotion.

The results highlight several directions for preventive action. First, interventions targeting young adults, particularly university students who are future parents, should promote awareness of the psychological consequences of corporal punishment and develop non-violent parenting skills. Second, public health programs should integrate gender-sensitive education that challenges stereotypes linking masculinity with control or toughness and reframes caregiving as compatible with emotional competence. Third, mental health and child protection systems should recognize young adults who still experience corporal punishment within families as a vulnerable population in need of support.

Overall, this study underscores that changing attitudes toward corporal punishment is a preventive strategy for enhancing youth wellbeing. By combining legal protection with sustained public education and gender-informed mental health promotion, societies can move toward eliminating corporal punishment and fostering healthier, more supportive environments for children and adolescents.

## Figures and Tables

**Table 1:** Results for the invariance tests related to the participant gender, parent gender and child gender.

Gender	Invariance	χ^2^/df/p	CFI	RMSEA [95%CI]	SRMR	Δχ^2^/Δdf/p
Participant	Free	28.75/54/0.998	1.000	0.000 [0.000–0.000]	0.030	
	Weak	78.16/62/0.081	0.998	0.030 [0.000–0.049]	0.047	49.41/8/0.000
	Strong	85.27/70/0.103	0.999	0.027 [0.000–0.046]	0.050	7.12/8/0.524
Parent	Free	30.79/54/0.995	1.000	0.000 [0.000–0.000]	0.030	
	Weak	50.15/62/0.860	1.000	0.000 [0.000–0.020]	0.038	19.37/8/0.013
	Strong	53.71/70/0.925	1.000	0.000 [0.000–0.011]	0.039	3.56/8/0.894
Child	Free	32.87/54/0.990	1.000	0.000 [0.000–0.000]	0.031	
	Weak	46.29/62/0.932	1.000	0.000 [0.000–0.010]	0.037	13.42/8/0.098
	Strong	49.73/70/0.968	1.000	0.000 [0.000–0.000]	0.038	3.44/8/0.904

**Table 2:** Descriptive statistics for items and total score.

Variable	M ^a^	SD ^b^	Min ^c^	Q1 ^d^	C ^e^	Q3 ^f^	Max ^g^	Skew ^h^	Kurt ^i^	SWp ^j^
Parent’s reaction is justified	3.4	1.45	1	2.0	4.0	5.0	5	−0.38	−1.24	<0.001
I am glad that the parent hit the child	2.6	1.52	1	1.0	2.0	4.0	5	0.43	−1.27	<0.001
If I was in the parent’s position, I would also apply corporal punishment	2.7	1.52	1	1.0	3.0	4.0	5	0.24	−1.41	<0.001
I think the child deserved the strike	3.4	1.51	1	2.0	4.0	5.0	5	−0.39	−1.32	<0.001
I am angry that the parent hit the child	2.6	1.33	1	1.0	2.0	4.0	5	0.39	−0.98	<0.001
If I were in the parent’s position, I would handle the problem by talking	3.7	1.24	1	3.0	4.0	5.0	5	−0.72	−0.45	<0.001
I think that the parent shouldn’t have hit the child	3.2	1.45	1	2.0	3.0	5.0	5	−0.12	−1.33	<0.001
I am satisfied with the parent’s reaction	2.7	1.39	1	1.0	3.0	4.0	5	0.23	−1.20	<0.001
If I were in the parent’s position, I would react more calmly	3.4	1.29	1	3.0	4.0	5.0	5	−0.45	−0.83	<0.001
ACP—scale score	2.9	1.19	1	1.9	2.8	3.9	5	0.12	−1.14	<0.001

^a^ mean; ^b^ standard deviation; ^c^ minimal recorded result; ^d^ first quartile; ^e^ median; ^f^ third quartile; ^g^ maximal recorded result; ^h^ skewness; ^i^ kurtosis; ^j^ *p*-value for Shapiro–Wilk test.

**Table 3:** ANCOVA model summary.

Effect	SS ^a^	df ^b^	MS ^c^	F ^d^	p	*η* ^2^	*η*ₚ^2^
Overall model	101.47	8	12.68	10.14	<0.001		
Previous CP experience	14.21	1	14.21	11.23	<0.001	0.017	0.019
Participant	56.81	1	56.81	44.87	<0.001	0.069	0.073
Parent	11.03	1	11.03	8.71	0.003	0.013	0.015
Child	14.88	1	14.88	11.75	<0.001	0.018	0.020
Participant: Parent	0.06	1	0.06	0.05	0.825	<0.001	<0.001
Participant: Child	3.79	1	3.79	2.99	0.084	0.005	0.005
Parent:Child	0.00	1	0.00	0.00	0.981	<0.001	<0.001
Participant: Parent:Child	0.69	1	0.69	0.55	0.460	<0.001	<0.001
Residuals	725.49	573	1.27				

^a^ sum of squares; ^b^ degrees of freedom; ^c^ mean squares; ^d^ F ratio; *η*^2^—eta squared; *η*ₚ^2^—partial eta squared.

**Table 4:** Descriptive statistics for the ANCOVA model effects and estimated marginal means corrected for previous experience with corporal punishment.

Variable	Group	N	Raw ^a^			EMM ^b^	
			M	SD	SE ^c^	M	SE
Participant gender	Male	291	3.18	1.180	0.069	3.13	0.068
	Female	291	2.57	1.131	0.066	2.50	0.068
Parent gender	Male	276	2.72	1.188	0.072	2.68	0.069
	Female	306	3.01	1.184	0.068	2.95	0.067
Child gender	Male	293	3.03	1.202	0.070	2.97	0.068
	Female	289	2.72	1.167	0.069	2.65	0.068
Previous CP experience	Yes	384	3.00	1.220	0.062		
	No	198	2.63	1.103	0.078		

^a^ raw descriptive statistics; ^b^ estimated marginal means; ^c^ standard error.

## Data Availability

Due to the sensitive nature of the data, the data are available upon request to the research team.

## Supplementary Materials

The following supporting information can be downloaded at: https://www.mdpi.com/article/10.3390/healthcare13233053/s1.

## Abbreviations

CP	Corporal punishment
SSS-CP	Short Situational Scale of Attitudes towards Corporal Punishment

## Appendix A. Short Situational Scale of Attitudes Towards Corporal Punishment (SSS-CP)

The text below presents the English translation of the vignette used to create the situation for the assessment of the attitude. Underlined parts are changed according to child’s and parent’s gender.

*Marko (16) is a student in the second year of high school in Karlovac. Since the beginning of high school, he has had several incidents mostly related to thefts and vandalism, but so far, there have been no contacts with the police or social services; everything has been resolved within the school. Last week, after school, his mother caught him counting money in his room. It was 800 HRK [Croatian Kuna], and she was certain that neither she nor the father had given it to him. She accused him of stealing the money, after which a quarrel ensued in which Marko told his mother she was lying and that she and his father never give him enough. He became enraged and called his mother “pathetic,” “disgusting,” and a “stupid peasant.” The mother reacted by slapping Marko across the face.*

What do you think about this event? Please mark your responses on the following questionnaire using the labels:

1—Strongly disagree

2—Somewhat disagree

3—Neither agree nor disagree

4—Somewhat agree

5—Strongly agree

1.	Mother’s reaction is justified	1	2	3	4	5
2.	I am glad that the mother hit Marko	1	2	3	4	5
3.	If I was in the mother’s position, I would also apply corporal punishment	1	2	3	4	5
4.	I think the Marko deserved the strike	1	2	3	4	5
5. *	I am angry that the mother hit Marko	1	2	3	4	5
6. *	If I were in the mother’s position, I would handle the problem by talking	1	2	3	4	5
7. *	I think that the mother shouldn’t have hit Marko	1	2	3	4	5
8.	I am satisfied with the mother’s reaction	1	2	3	4	5
9. *	If I were in the mother’s position, I would react more calmly	1	2	3	4	5
Starred items should be reverse coded.						
