# Supplementary material for: Gendered Attitudes Toward Corporal Punishment: Implications for Prevention of Mental Health Problems in Youth
Source: Healthcare (Basel). 2025 Nov 25;13(23):3053. doi: 10.3390/healthcare13233053 (PMC12692408; doi:10.3390/healthcare13233053)
Supplement: Supplementary file 1 [file healthcare-13-03053-s001.zip › healthcare-3959317-supplementary.pdf]

R QUARTO CODE

````
---
title: "Gender aspects of attitudes towards corporal punishment of
children"
author:
  - name: "Miroslav Rajter"
    affiliation: "University of Zagreb, Faculty of Law"
  - name: "Milani Medvidović"
    affiliation: "elektrotehnička škola - Split"
toc: true
number-sections: true
execute:
  echo: false
format:
  html:
    theme: default
    code-fold: true
editor_options:
  markdown:
    wrap: 72
---

# Introduction

This document presents all analyses conducted in the creation of the
article. All analyses were performed using R 4.1.2 [@rcoreteam2021] with
*tidyverse* package [@wickham2019]. The confirmatory factor analyses
were performed using *lavaan* package [@rosseel2012].

# Data preparation

The data was downloaded from GitHub and placed in _df_ variable. The data
has defined variable and value labels that can be accessed with
_sjlabelled_ package.

```{r}
#| cache: false
#| warning: false
#| message: false
library(tidyverse)
library(lavaan)
df=readr::read_rds("https://github.com/mrajter/acp_paper/raw/main/data_file
.rds")


```

## Variable definition

The variables in the dataset are coded as follows:

-   rbi - participant code

-   part_gen - participant's gender (F>M)

-   par_gen - parent's gender (vignette variant) (F>M)
````

- ch_gen - child's gender (vignette variant) (F>M)

- age - age of the participant

- cp_exp - experience with being corporally punished as a child

- acp1 - acp9 - items for the Attitudes towards Corporal Punishment scale

CFA

Basic CFA

The presumed structure for the Attitudes towards Corporal Punishment - Short Situational Scale was single factor solution. The model was assessed using *lavaan* package using diagonally weighted least squares estimator that is more appropriate to the data[@li2016]. The model showed an excellent theoretical fit and the standardized estimates with model fit indices are shown below.

```{r}
#| cache: true

#function for model fit assessment
cp.fm=function(fit){
  a=fitmeasures(fit,fit.measures=c("chisq", "df", "pvalue", "cfi", "rmsea",
"rmsea.ci.lower", "rmsea.ci.upper", "srmr"))
  b=list()
  b$chisq=format(round(a[1],2), nsmall=2)
  b$df=format(round(a[2],0), nsmall=0)
  b$p=format(round(a[3],3), nsmall=3)
  b$cfi=format(round(a[4],3), nsmall=3)
  b$rmsea=paste0(format(round(a[5],3), nsmall=3), "[",
format(round(a[6],3), nsmall=3), "-", format(round(a[7],3), nsmall=3), "]")
  b$srmr=format(round(a[8],3), nsmall=3)
  return(b)
  }



model='
att_cp =~ acp1 + acp2 + acp3 + acp4 + acp5 + acp6 + acp7 + acp8 + acp9
'
fit=cfa(model= model, data=df, estimator="DWLS")
#not shown for brevity. For a detailed view remove comments below
#cp.fm(fit)
#summary(fit, fit.measures=T,standardized=TRUE)
```

Measurement invariance

The measurement invariance was assessed for three variables related to

the participant gender, parent gender and child gender. The primary method of the invariance testing was to create models for each group (*group* parameter in *cfa* function) and then to use constraints for loadings and intercepts. The invariance was assessed by the difference in χ^2 provided by *anova* function and changes in *cfi*, *rmsea* and *srmr* parameters. The function for creating the table with the fit parameters is in the code below.

```{r}
#| cache: true

invariance.fit=function(f1,f2,f3){

cp.fm0=cp.fm(f1)
for.table0=c( #original
  paste0(cp.fm0$chisq,"/",cp.fm0$df, "/", cp.fm0$p),
  cp.fm0$cfi,
  cp.fm0$rmsea,
  cp.fm0$srmr,
  NA)

a1=anova(f1,f2)
cp.fm1=cp.fm(f2)
#weak
for.table1=c(
  paste0(cp.fm1$chisq,"/",cp.fm1$df, "/", cp.fm1$p),
  cp.fm1$cfi,
  cp.fm1$rmsea,
  cp.fm1$srmr,
  paste0(
    format(round(a1$`Chisq diff`[2], 2), nsmall=2),
    "/",
    a1$`Df diff`[2],
    "/",
    format(round(a1$`Pr(>Chisq)`[2],3), nsmall=3)
    )
  )
#strong
a2=anova(f2,f3)
cp.fm2=cp.fm(f3)
for.table2=c( #weak
  paste0(cp.fm2$chisq,"/",cp.fm2$df, "/", cp.fm2$p),
  cp.fm2$cfi,
  cp.fm2$rmsea,
  cp.fm2$srmr,
  paste0(format(round(a2$`Chisq diff`[2], 2), nsmall=2), "/", a2$`Df
diff`[2],"/",  format(round(a2$`Pr(>Chisq)`[2],3), nsmall=3))
)

res=data.frame(Invariance=c("Free", "Weak", "Strong"))
res=cbind(res, rbind(for.table0, for.table1, for.table2))
rownames(res)=NULL

result=list()
result$df=res

r=flextable::flextable(res) %>%
```

```
  flextable::set_header_labels(
    values=list(
      Invariance="Invariance",
      V1=paste0("\U1D6D8", "\U00B2", "/df/p"),
      cfi="cfi",
      V3="rmsea[95%CI]",
      srmr="srmr",
      V5=paste0("\U0394", "\U1D6D8", "\U00B2", "/", "\U0394", "df/p")
  )) %>%
  flextable::align(align="center", part="all") %>%
  flextable::autofit()

result$table=r

colnames(result$df)=c("Invariance", "chi_sq", "cfi", "rmsea", "srmr",
"delta_chi_sq")

return(result)
}

```

Participant gender

```{r}
#| cache: true
fit_part1=cfa(
  model= model,
  data=df,
  estimator="DWLS",
  group="part_gen")

fit_part2=cfa(
  model= model,
  data=df,
  estimator="DWLS",
  group="part_gen",
  group.equal=c("loadings"))

fit_part3=cfa(
  model= model,
  data=df,
  estimator="DWLS",
  group="part_gen",
  group.equal=c("loadings", "intercepts"))

invariance.fit(fit_part1, fit_part2, fit_part3)$table




```

The results show that all three models fit the data well. Although the difference in the models is statistically significant based on the χ^2 tests, the difference in *cfi* parameter is less than 0.002, which is the most strict cut-off value as recommended by Kline

[-@kline2016, p. 401]. Regarding the χ^2 tests it has to be noted that the model fit indices based on the χ^2 tests show adequate fit of the each model to the data.

Parent gender

The same method was applied to the parent gender variable.

```{r}
fit_par1=cfa(
  model= model,
  data=df,
  estimator="DWLS",
  group="par_gen")

fit_par2=cfa(
  model= model,
  data=df,
  estimator="DWLS",
  group="par_gen",
  group.equal=c("loadings"))

fit_par3=cfa(
  model= model,
  data=df,
  estimator="DWLS",
  group="par_gen",
  group.equal=c("loadings", "intercepts"))

invariance.fit(fit_par1, fit_par2, fit_par3)$table
```

The results show that the parameter changes for the approximate fit indexes are not supporting the rejection of invariance.

Child gender

The final invariance test was conducted for the child gender.

```{r}
fit_ch1=cfa(
  model= model,
  data=df,
  estimator="DWLS",
  group="ch_gen")

fit_ch2=cfa(
  model= model,
  data=df,
  estimator="DWLS",
  group="ch_gen",
  group.equal=c("loadings"))

fit_ch3=cfa(
  model= model,
  data=df,
  estimator="DWLS",
```

  group="ch_gen",
  group.equal=c("loadings", "intercepts"))

invariance.fit(fit_ch1, fit_ch2, fit_ch3)$table
```

The results show no significant changes between models, even when using the $\chi^2$ tests.

We can conclude that the Attitudes towards Coproral Punishment - Short Situational Scale is functionally invariant regarding the participant gender and the parent's and child's gender described in the vignette.

## Scale score calculation

The final step was to assess the method for the creation of the total scale score. The preferred method was to use average result (mean score) on the scale items. This includes the reversal of items 5, 6, 7 and 9. This score was compared to the factor scores as predicted with CFA.

```{r}

#factor scores
df=cbind(df, lavaan::predict(fit))

# calculation of total score as items mean

# function for item reversal
rev.code=function(item){
  item=(item-6)*-1
  return(item)
}


df$acp5_r=rev.code(df$acp5)
df$acp6_r=rev.code(df$acp6)
df$acp7_r=rev.code(df$acp7)
df$acp9_r=rev.code(df$acp9)

#calculate total score as row mean
df$acp_mean=rowMeans(df%>%select(acp1, acp2, acp3, acp4, acp5_r, acp6_r,
acp7_r, acp8, acp9_r))

#correlation of item means with factor scores
score_cor=cor.test(df$att_cp, df$acp_mean)

#calculate cronbach alpha
scale.alpha=psych::alpha(df %>% select(acp1, acp2, acp3, acp4, acp5_r,
acp6_r, acp7_r, acp8, acp9_r))
scale.alpha=substr(format(round(unname(scale.alpha$total[1]), 3),
nsmall=3), 2,5)
```

The results show that the correlation of scores calculated as items mean (
_acp_mean_)
and the factor scores (_att_cp_) is `r format(round(score_cor$estimate,
3),
```

nsmall=3)`, thus indicating that the items mean as the scale score calculation method is functionally equal to factor scores and because of practical use the preferred method for the total score calculation. The Cronbach $\alpha$ reliability of the scale is `r scale.alpha`.

Item and scale descriptives

The descriptive statistics for items and scale is shown in the table below. Ste descriptive statistics were calculated using _reflexR_ package [@rajter2022] and the chart was made using _ggplot2_ [@wickham2016] and _jtools_ [@long2022].

````
```{r}
#add variable label to acp_mean
df$acp_mean=sjlabelled::set_label(df$acp_mean, "ACP - scale score")


#set up options for reflexR
reflexR.opts=list()
reflexR.opts$lang="en"
reflexR.opts$d.p="."
reflexR.opts$lead.zero=F
reflexR.opts$p.type="<>"

# descriptives
t=reflexR::des.flex(df, 1~acp1 + acp2 + acp3 + acp4 + acp5 + acp6 + acp7 +
acp8 + acp9 + acp_mean, option=reflexR.opts)
t$table=flextable::set_caption(t$table, "Descriptive statistics for items
and total score")
t$table=flextable::footnote(t$table,
                            i=1, j=2:13,
                            value = flextable::as_paragraph(
                              c("number of valid results",
                                "mean",
                                "standard deviation",
                                "minimal recorded result",
                                "first quartile",
                                "median",
                                "third quartile",
                                "maximal recorded result",
                                "skewness",
                                "kurtosis",
                                "Shapiro-Wilk test",
                                "p-value for Shapiro-Wilk test")
                            ),
                            ref_symbols = c("a", "b", "c", "d", "e", "f",
"g", "h", "i", "j", "k", "l"),
                            part="header",
                            inline=TRUE
                            )
t$table
```
````

The results show that the answers of participants are on average close to the theoretical mean of the scale which gives a good indication of the sensitivity of the instrument. All of the analyzed variables were significantly different from the normal distribution. Although the absolute

values of skewness were relatively small, indicating a good scattering of the participants throughout the scale, values of kurtosis indicate a somewhat "flatter" distribution, as shown in the figure below, which is to be expected when the attitudes are measured on the controversial topic.

````
```{r}
library(ggplot2)
ggplot(df, aes(x=acp_mean)) +
 geom_histogram(aes(y=..density..), colour="black", fill="white", bins=20)+
 geom_density(alpha=.2, fill="#FF6666") +
  xlab("Attitude towards corporal punishment") +
  ylim(0,1) +
  jtools::theme_apa()
#ggsave(filename="histogram.tiff", path = "pics/", scale=1, device='tiff',
dpi=700)
```
````

The histogram shows that the distribution of results has more density in the lower part of the scale, indicating a somewhat more negative attitude and somewhat more grouping on the scale center, however those differences are without extreme groupings.

ANOVA

The final analysis was focused on testing the differences regarding the participant, parent and child gender. Three-way ANCOVA was performed with main effects of participant, parent and child gender. All interaction effects were also analyzed analyzed and the previous experience with being corporally punished was used as a covariate. The analysis was performed using type III sum of squares method with _jmv_ package [@selker2022]. The tables below show the model summary and the descriptive statistics for the significant effects.

````
```{r}
#ancova
df$part_gen=as.factor(df$part_gen)
df$par_gen=as.factor(df$par_gen)
df$ch_gen=as.factor(df$ch_gen)
q=jmv::ancova(
  formula = acp_mean ~ cp_exp + part_gen + par_gen + ch_gen +
part_gen:par_gen + part_gen:ch_gen + par_gen:ch_gen +
part_gen:par_gen:ch_gen,
  data = df,
  effectSize = c("eta", "partEta"),
  modelTest = TRUE,
  homo = TRUE,
  emMeans = ~ part_gen + par_gen + ch_gen,
  emmPlots = FALSE,
  emmTables = TRUE)

q.df=q$main$asDF
q.df$name=c(
  "Overall model",
  "Previous CP experience",
  "Participant",
  "Parent",
  "Child",
````
````

````
  "Participant:Parent",
  "Participant:Child",
  "Parent:Child",
  "Participant:Parent:Child",
  "Residuals")

rownames(q.df)=NULL

#helper function for transforming pvals
p.val.apa.trans=function(num){
  if (is.na(num)) {return(NA)}
  num2=format(round(abs(num),3), nsmall=3)
  num2=substr(num2, 2,5)
  if(abs(num)==1) {num2="1.000"}
  if (num<0) {num2=paste0("-",num2)}
  if (abs(num)<0.001) {num2="<.001"}
  return(num2)
}


q.df=q.df %>% mutate(
  ss=round(ss,2),
  ms=round(ms,2),
  F=round(F,2),
  p=unlist(lapply(p, p.val.apa.trans)),
  etaSq=unlist(lapply(etaSq, p.val.apa.trans)),
  etaSqP=unlist(lapply(etaSqP, p.val.apa.trans))
)

std_border = officer::fp_border(color="black", width = 1)

q.table=q.df %>%
  flextable::flextable() %>%
  flextable::set_header_labels(values=list(
    name="Effect",
    ss="SS",
    df="df",
    ms="MS",
    F="F",
    p="p",
    etaSq=paste0("\U1D702", "\U00B2"),
    etaSqP=paste0("\U1D702", "\U209A\U00B2")
  )) %>%
  flextable::align(j=2:ncol(q.df), align = "center", part="body") %>%
  flextable::align(align = "center", part="header") %>%
  flextable::hline_top(border=std_border, part="header") %>%
  flextable::hline(i=1, border=std_border, part="header") %>%
  flextable::hline_bottom(border=std_border, part="body") %>%
  flextable::set_caption("ANCOVA model summary")
q.table

```



```{r}
#helper function for raw descriptives
ancova.desc.raw=function(vari, df_t=df){
````

```
  df_t1=df_t %>% filter((.)[vari]==0)
  df_t2=df_t %>% filter((.)[vari]==1)
  N=c(
    nrow(df_t1),
    nrow(df_t2))
  M=c(
    round(mean(df_t1$acp_mean),2),
    round(mean(df_t2$acp_mean),2))
  SD=c(
    round(sd(df_t1$acp_mean),3),
    round(sd(df_t2$acp_mean),3))
  SE=c(
    round(SD[1]/sqrt(N[1]),3),
    round(SD[2]/sqrt(N[2]),3))

return(cbind(N,M,SD, SE))
}

desc.anc=rbind(
  ancova.desc.raw("part_gen"),
  ancova.desc.raw("par_gen"),
  ancova.desc.raw("ch_gen"),
  ancova.desc.raw("cp_exp")
)

#get EMM's

desc.emm=rbind(
  q$emm[[1]]$emmTable$asDF[,2:3],
  q$emm[[2]]$emmTable$asDF[,2:3],
  q$emm[[3]]$emmTable$asDF[,2:3])
desc.emm$mean=round(desc.emm$mean,2)
desc.emm$se=round(desc.emm$se,3)
desc.emm=rbind(desc.emm, c(NA,NA), c(NA,NA))

#full table
desc.anc=cbind(
  data.frame(
    Variable=c(
      "Participant gender",
      "Participant gender",
      "Parent gender",
      "Parent gender",
      "Child gender",
      "Child gender",
      "Previous CP experience",
      "Previous CP experience"
    ),
    Group=c(
      "Male",
      "Female",
      "Male",
      "Female",
      "Male",
      "Female",
      "Yes",
      "No"
```

```
    )
    ),
  desc.anc,
  desc.emm
)

#to table
desc.anc %>%
  flextable::flextable()  %>%
  flextable::set_header_labels(values=list(
    Variable="Variable",
    Group="Group",
    N="N",
    M="M",
    SD="SD",
    SE="SE",
    mean="M",
    se="SE"
  )) %>%
  flextable::add_header_row(values=list(
    Variable="Variable",
    Group="Group",
    N="N",
    M="Raw",
    SD="Raw",
    SE="Raw",
    mean="EMM",
    se="EMM"
  )) %>%
  flextable::align(j=3:8, align="center", part="body") %>%
  flextable::align(align="center", part="header") %>%
  flextable::merge_h(i=1, part="header") %>%
  flextable::merge_v(j=1:3, part="header") %>%
  flextable::merge_v(j=1, part="body") %>%
  flextable::hline(i=1, j=4:8, border=std_border, part="header") %>%
  flextable::hline(i=2, border=std_border, part="header") %>%
  flextable::hline(i=2, border=std_border, part="body") %>%
  flextable::hline(i=4, border=std_border, part="body") %>%
  flextable::hline(i=6, border=std_border, part="body") %>%
  flextable::hline(i=8, border=std_border, part="body") %>%
  flextable::hline_top(part="header") %>%
  flextable::vline(j=6, border=std_border, part="all") %>%
  flextable::fix_border_issues() %>%
  flextable::set_caption("Descriptive statistisc for the ANCOVA model
effects and estimated marginal means corrected for previous experience with
coproral punishment")
desc.anc
```

The results show that all three main effects are statistically significant and none of the interaction effects are statistically significant. In terms of the effect sizes, the most important effect is participant that explains 6.8% (η^2) of the attitudes towards corporal punishment with males having more positive attitude than females. Other two main effects explain less than 2% of the dependent variable and show that the participants have more positive attitude towards corporal punishment when the perpetrator is

female and the child is male. The interaction effects were insignificant and in terms of effect sizes explained a trivial amount of the variance of the dependent variable.
